# Undernutrition and 60-day mortality in critically ill children with respiratory failure: a prospective cohort study

**DOI:** 10.1186/s12887-023-04087-x

**Published:** 2023-05-30

**Authors:** Sina Amirjani, Narjes Ahmadizadeh, Azita Behzad, Yasaman Dadashi-noshahr, Zahra Vahdat Shariatpanahi

**Affiliations:** 1 Department of Clinical Nutrition and Dietetics, Faculty of Nutrition and Food Technology, National Nutrition and Food Technology Research Institute, Shahid Beheshti University of Medical Sciences, Tehran, Iran; 2 Department of Critical care, Mofid children’s hospital, Faculty of Medicine, Shahid Beheshti University of Medical Sciences, Tehran, Iran; 3 Address: Farahzadi Blvd., Shahrak Qods, 1981619573 Baran, Tehran, West Arghavan PO Box: 19395-4741, Iran

**Keywords:** Nutritional status, Mortality, Underfeeding, PIM2

## Abstract

**Background:**

To determine whether undernutrition affects 60-day mortality in pediatric acute respiratory failure.

**Methods:**

Subjects with acute respiratory failure aged between two months and 13 years were included in the study. The Z-scores were calculated on admission and children were categorized into two groups of undernutrition and normal nutrition. The nutritional intake of the children was measured daily. The outcome was 60-day mortality.

**Results:**

A total of 126 patients met the inclusion criteria; 41% were undernourished based on the Z-score of BMI and weight for height, 50% based on the Z-score of height and length for age and 45% based on the Z-score of weight for age. Overall, the 60-day mortality rate was 27.8%. The Cox regression analysis adjusted with PIM2, age and gender, showed that undernutrition has a significant relationship with 60-day mortality based on the weight for age Z-score (HR = 2.33; CI: 1.175–4.638). In addition, undernutrition has a significant relationship with 60-day mortality based on the BMI for age (HR = 3.04; CI:1.070–8.639) and weight for height (HR = 2.62; CI: 1.605–6.658) Z-scores. The mean calorie and protein intake of 72% of the children was less than 80% of their calorie needs. The time to start feeding in 63% of the children was more than 48 h. There was no relationship between the time of starting nutrition and nutritional intake during PICU admission and mortality.

**Conclusion:**

Undernutrition is prevalent in mechanically ventilated children in the PICU and may be associated with 60-day mortality.

## Introduction

According to the European Society of Parenteral and Enteral Nutrition (ESPEN), malnutrition is a state in which subnormal intake or uptake of nutrients results in impaired physical and mental function. Moreover, malnutrition causes suboptimal clinical disease outcome [1].

Malnutrition is common on admission to pediatric intensive care units (PICUs) with a prevalence of 15–65%.[2].

These children require additional nutrition care since they are more vulnerable to complications resulting from malnutrition [3]. Previous studies have reported that malnourished children admitted to the PICU are at a greater risk of mortality and other adverse outcomes such as infection and prolonged PICU stay [4, 5].

The high prevalence of malnutrition in children admitted to the PICU is caused by many factors, such as the high prevalence of chronic diseases in these children. Moreover, as the duration of PICU stay increases, the nutritional state of these children declines due to factors such as decreased intake of protein and increased protein catabolism [6–8].

Given the detrimental effects of malnutrition in children admitted to the PICU and the high prevalence of malnutrition in these children, it is recommended that all children undergo nutritional assessment within 48 h of admission to this unit [9]. Different methods can be used to screen and identify children with malnutrition or those who are at risk of undernutrition. The prevalence of malnutrition based on the weight-for-age values varies depending on the location and target population and has been reported up to 50% in previous studies [9, 10].

Acute respiratory failure, a state in which O2 provision and CO2 removal do not match the body’s metabolic demand, is one of the major causes of PICU admission. Although respiratory failure is diagnosed by blood gas abnormalities, the management of this condition mainly depends on the underlying pathophysiology. The most common cause of respiratory failure is disorders involving the respiratory tract. Nonetheless, there are many other states that can result in respiratory failure, such as mechanical impairment of ventilation, failure of central nervous system and metabolic disorders [11, 12]. Undernutrition negatively affects the respiratory system by decreasing the function of the respiratory muscle and diaphragm and changing the lung structure, the immune function and ventilatory control, all of which predispose the patient to respiratory failure and long-term mechanical ventilation. Malnutrition at admission, based on anthropometric indicators, has long been shown to be a predictor of outcomes in mixed and specific groups of critically ill pediatric patients [13–20].

According to observational studies, PICU guidelines suggest early initiation of enteral nutrition within the first 24–48 h after PICU admission in eligible patients [9, 21]. Regarding target energy intake in critically ill children, guidelines recommend achieving at least two-thirds of the daily energy requirement by the end of the first week in the PICU, based on observational cohort studies [9]. Many of the recommendations or suggestions in guidelines for the provision and evaluation of nutritional support therapy in the pediatric critically ill patient are driven by consensus or low-level evidence [9] and there is a lack of high-quality evidence to guide nutrition in acute pediatric illnesses [21]. These guidelines therefore emphasizes that future studies must examine the optimal energy dose that is associated with improved nutrition and clinical outcomes in critically ill children and future studies are also needed to determine the optimal dose of protein that improves relevant clinical outcomes [9, 21].

In line with this goal and since no study in our country has been conducted in this regard, the present study was conducted with the aim of investigating the relationship between undernutrition based on Z-scores (BMI for age, weight for age, and weight for length) and protein and energy intake with 60-day mortality in critically ill children with acute respiratory failure.

## Methods

### Study population and design

This was a single center, prospective cohort study carried out at the PICU of Mofid Children’s Hospital. The study was approved by an appropriate institution and all methods were performed in accordance with the relevant guidelines and regulations. The children between 2 months and 13 years old who were admitted to the PICU due to respiratory failure and had not participated in other nutritional studies over the last 30 days were screened for the eligibility criteria. The exclusion criteria were as follows: Duration of PICU stay less than 76 h and having been diagnosed with cancer or covid-19. In addition, patients who were overweight and obese based on their anthropometric parameters (BMI Z-score > 1) were not included in this study.

### Data collection

Over the first 24 h after admission to the PICU, the pediatric index of mortality 2 (PIM2) was measured for all the children to assess their disease severity. PIM2 is a scoring system that predicts the risk of death for pediatric patients admitted to intensive care units. The score is calculated using 12 variables that include age, sex, admission diagnosis, and physiological measurements. Higher scores indicate a higher risk of mortality [22].

The children’s demographic characteristics were also collected from their medical records at the day of admission.

Anthropometric data were collected by a trained nurse. In cases where it was not possible to measure, weight and height data were obtained from medical records. The Z-scores of BMI, height. and weight for age of the children > 2 years old were calculated based on the Centers for Disease Control and Prevention (CDC) formula. For children younger than 2 years, the Z-scores of weight for age, length for age and weight for length were calculated based on the World Health Organization (WHO) formula. The children were categorized into two groups: Undernutrition (Z-score < -2) and normal nutrition (Z-score ≥ -2 and ≤ 1). Moreover, the children were categorized into two groups based on the time of start of nutrition (> 48 h, ≤ 48 h).

The nutritional intake of the children was measured by a clinical nutritionist every day during the PICU stay. Their daily nutritional needs were also calculated. The energy requirement of the children was estimated by the Schofield equation and 1.5 g of protein per kilogram of body weight was regarded to be sufficient, and the nutritional intake of the children was compared against these values [9].

Based on their nutritional intake, the children were categorized into two group: Suboptimal intake (< 80% of the energy and protein needs) and optimal intake (≥ 80% of the energy and protein needs).

The main outcome of this study was to evaluate the impact of nutritional state and intake on 60-day mortality of children admitted to the PICU due to respiratory failure.

### Statistical analysis

Data were analyzed using SPSS version 21 statistical software. In this study, P-values less than 0.05 were considered statistically significant. Data are shown as mean ± standard deviation or median (interquartile range or IQR) based on variable type and frequency (percentage) for the qualitative variables. The normality of the data was evaluated using the Kolmogorov-Smirnov test. In the case of normal distribution, parametric tests were used, and in the case of non-normal distribution, non-parametric tests were used.

In order to compare the qualitative variables between the two groups, the Chi-square test was implemented. Also, to compare the mean of the quantitative variables between the groups, if the distribution of the variable was normal, the T test was used, and if the distribution of the variable was not normal, Mann-Whitney’s test was administered.

The Cox proportional hazards model was used to estimate the hazard of mortality with undernutrition with and without adjustments for admission diagnosis, PIM2 and age. We applied Kaplan-Meier curves and a log-rank test for the estimation and comparison of crude survival between the patients in the two categories of undernutrition and normal nutrition.

The sample size was 110 people based on the information of a previous study in which the mortality rate in subjects without undernutrition was 10% and in subjects with undernutrition was 27% with hazard ratio for mortality of 3.75 [10].

## Results

**Fig. 1:**
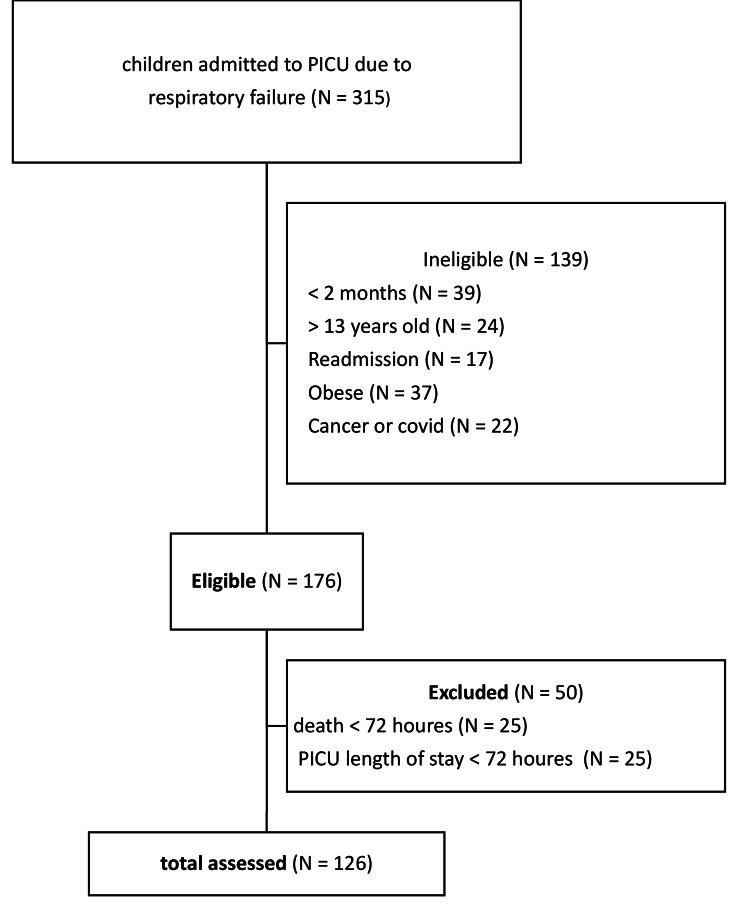
Flowchart of subjects

A total of 315 children were admitted to the surveyed PICU between august 2021 to august 2022 due to respiratory failure. A total of 139 of them did not meet the inclusion criteria (< 2 months old: n = 39, > 13 years old: n = 24, readmission: n = 17, obese: n = 37, cancer or covid: n = 22). Therefore, 176 critically ill children were included in the study. Out of them, 50 children were excluded due to PICU length of stay or death < 72 h. Ultimately, the study was conducted on 126 patients. The flow chart of the participants is shown in Fig. 1.

### Baseline characteristics

Table 1 presents the baseline characteristics of the 126 patients included in this study. A total of 59.5% of them were male (n = 75). The median age was 24 months (IQR = 60). Based on the Z-score of weight for age, 45.2% of the patients were considered undernourished (n = 57). Based on the Z-score of height and length for age, 50% of the patients were considered undernourished (n = 63). For the children above 2 years old, the prevalence of undernutrition was also calculated according to the Z-score of BMI for age, and for the children younger than 2 years old, this rate was measured based on the Z-score of weight for length and a total of 41% of the children were found to be underweight (n = 28 in ≥ 2 years old, n = 24 in < 2 years old).

**Table 1:** Baseline and nutritional characteristics of patients

Characteristics	Value (126 patients)
Sex (male), N (%)	75 (59.5%)
Age, months (Median, IQR)	24.00 (60.00)
PIM2 (Mean, SD)	6.25 (8.83)
BMI for age, z score, N (%)	
Well-nourished Undernourished	30 (51.7) 28 (48.3)
Weight for age, z score, N (%)	
Well-nourished Undernourished	69 (54.8) 57 (45.2)
Height, Length for age, z score, N (%)	
Well-nourished Undernourished	63 (50) 63 (50)
Weight for length, N (%)	
Well-nourished undernourished	44 (64.7) 24 (35.3)
BMI for age (> 2 years) or Weight for length (< 2years), N (%)	
Well-nourished Undernourished	75 (59.5) 51 (40.5)
Energy intake, N (%)	
< 80% of needs > 80% of needs	90 (71.4) 36 (28.6)
Protein intake, N (%)	
< 80% of needs > 80% of needs	96 (76.2) 30 (23.8)
Starting Feeding, N (%)	
< 48 h > 48 h	47 (37.3) 79 (62.7)
Admitting diagnosis, N (%)	N (%)
Pneumonia Cystic Fibrosis Neurological disorder Congenital heart disease Gastrointestinal disorder Trauma	19 (15) 56 (44) 31 (24) 8 (6) 5 (4) 7 (6)

BMI, body mass index; PIM2, Pediatric Index of Mortality 2

Demographic and clinical outcomes of children based on Z-scores and mean calorie/protein intake are shown in Tables 2 and 3.

**Table 2:** Demographic and clinical outcomes of children based on Z-scores

Characteristics	Wt for age ≥ -2 (N = 69)	Wt for age < -2 (N = 57)	P value	Wt/Lt ≥ -2 and BMI ≥ -2 (N = 75)	Wt/Lt <-2 and BMI<-2 (N = 51)	p-value	Ht, Lt/age, ≥-2 (N = 63)	Ht, Lt/age, <-2 (N = 63)	p-value
Sex (male)*	43 (62)	32 (56)	0.585	44 (58)	31 (60)	0.855	25 (40)	38 (60)	0.10
Age **	2 (4)	2 (6.34)	0.865	2 (5)	2 (4.34)	0.759	2 (5)	2 (4.34)	0.23
PIM2***	6.3 (8.3)	6.2 (11.5)	0.483	6.3 (8.4)	6 (8.8)	0.773	7.74 (7.3)	6.4 (6.0)	0.61
PICU stay***	8 (10)	8 (10)	0.883	8 (9)	10 (10)	0.453	10.7 (9.5)	12.6 (9.7)	0.48
Mortality*	13 (18)	22 (38)	0.012	12 (16)	23 (45)	0.001	17(27)	18 (28)	0.9

Wt, Weight; Lt, Length; Ht, Height; BMI, body mass index; PIM2, Pediatric Index of Mortality 2.

* N (%), ** median (IQR), *** (Mean, SD)

**Table 3:** Demographic and clinical outcomes of children among nutritional status categories based on start of feeding time

Characteristics	Feeding > 48 h (N = 47)	Feeding ≤ 48 h (N = 79)	p-value
Sex (male) *	30 (63)	45 (56)	0.448
Age**	2 (3.42)	2 (5)	0.185
PIM2***	4.55 (8.55)	6.7 (7.9)	0.234
PICU stay, day***	8 (14)	8 (10)	0.248
Mortality*	12 (25)	23 (29)	0.664

BMI, body mass index; PIM2, Pediatric Index of Mortality 2.

N (%), ** median (IQR), *** (Mean, SD)

### Nutritional intake

All the children were on enteral nutrition via nasogastric tube. The mean calorie intake of 72% of the children during hospitalization was less than 80% of their calorie needs. 76% of the children received lower than 80% of their protein requirement. The time to start feeding was less than 48 h in 37.3% of the children admitted to the PICU (Table 1).

### Clinical outcomes

The 60-day mortality was 27.8% (n = 35) in this study and there was a significant difference (p = 0.012) in the mortality rate between the malnourished and well-nourished groups based on the weight-for-age Z-score (Table 2). In the well-nourished group, 18% (n = 13) of children died, while in the malnourished group, this rate was 38% (n = 22). As shown in Table 2, the incidence of mortality in malnourished individuals (45%, n = 23) based on the Z-scores of weight for length (for children under 2 years of age) and BMI for age (for children over 2 years of age) was significantly higher (p < 0.001) than individuals in the well-nourished group (16%, n = 12). This rate was not significant between the two groups of malnourished and well-nourished based on Z-score of length and height for age.

According to Tables 3 and 4, the mortality rate was not significantly different based on the average intake of energy and protein or the time of starting feeding.

**Table 4:** Demographic and clinical outcomes of children based on protein and energy intake

Characteristics	Calorie intake ≥ 80% (N = 36)	Calorie intake < 80% (N = 90)	p-value	Protein intake ≥ 80% (N = 30)	Protein intake < 80% (N = 96)	p-value
Sex (male) *	23 (63)	52 (57)	0.554	16 (53)	59 (61)	0.429
Age**	2.5 (6.4)	2 (4)	0.948	2 (5.25)	2 (5.19)	0.993
PIM2***	5.98 (11.6)	6.25 (8.5)	0.576	5.02 (13.2)	6.45 (8.65)	0.659
PICU stay, day***	17.5 (22)	7 (7)	< 0.001	7 (7)	17.5 (22)	< 0.001
Mortality*	9 (25)	26 (28)	0.826	8 (26)	27 (28)	0.538

BMI, body mass index; PIM2, Pediatric Index of Mortality 2.

N (%), ** median (IQR), *** (Mean, SD)

Table 5 shows the risk factors of 60-day mortality. The Cox regression analysis showed that undernutrition has a significant relationship with mortality based on weight for age (HR = 2.626) and BMI for age (HR = 3.3) Z-scores. Nevertheless, the weight for length Z-score, the height and length for age Z-score, the time of starting to feed and the intake of energy and protein had no significant relationship with mortality rate.

**Table 5:** Risk factors for 60-day mortality in under-nourished children

Variables	Unadjusted analysis		Adjusted analysis*	
	Hazard ratio (95% CI)	P value	Hazard ratio (95% CI)	P value
BMI for age, Z score < -2	3.300 (1.137–9.579)	0.028	3.041 (1.070–8.639)	0.038
Weight for age, Z score < -2	2.626 (1.286–5.364)	0.008	2.335 (1.175–4.638)	0.015
Height/Length for age, Z score < -2	1.030 (0.441–1.981)	0.766	1.101 (0.678–2.123)	0.671
Weight for length < -2	2.523 (0.931–6.833)	0.069	2.626 (1.605–6.658)	0.042
BMI for age (> 2 years) and Weight for length (< 2years), Z score < -2	3.293 (1.637–6.627)	0.001	3.269 (1.034–6.667)	0.001
Energy intake < 80% of needs	0.757 (0.351–1.633)	0.478	0.760 (0.356–1.623)	0.478
Protein intake < 80% of needs	0.837 (0.377–1.856)	0.661	0.848 (0.385–1.866)	0.682
Start feeding ≥ 48 h	1.057 (0.514–2.172)	0.880	1.210 (0.602–2.432)	0.592

* Adjusted by age, admission diagnosis. and PIM2

After adjusting the Cox regression analyses with PIM2, age and admission diagnosis, the relationship between weight for age and BMI for age (≥ 2 years) remained significant and the significance of the relationship between mortality and the Z-score of weight for length changed and became significant (HR = 2.626).

**Fig. 2:**
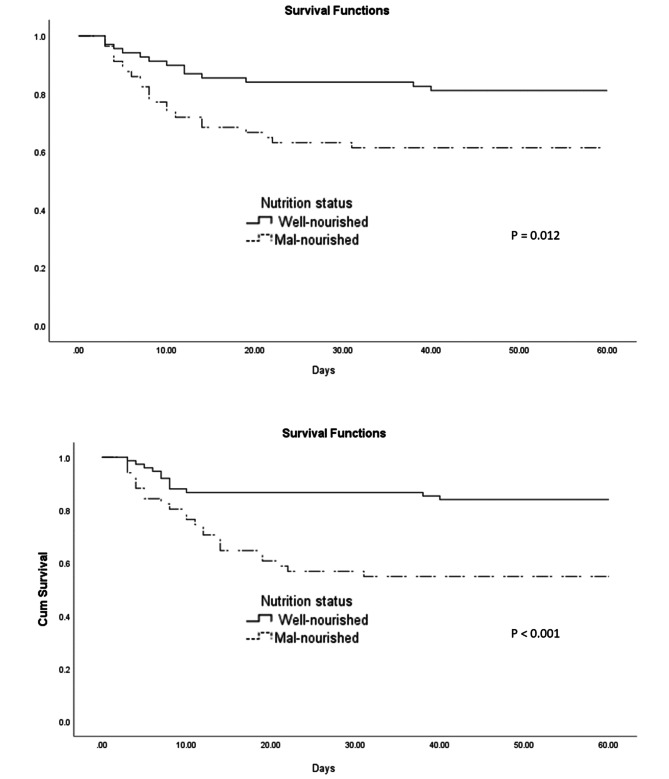
Survival rates of participants compared between two groups of nutritional status based on Z score BMI for age and weight for length (upper) and weight for age (lower)

Kaplan-Meier curve (Fig. 2) showed that survival was better in the well-nourished patients than that in the malnourished based on BMI for age / weight for length Z scores [39.72 days (36.76–42.67) vs. 29.70 days (24.88–34.52), P < 0.001; log rank] and weight for age Z score [39.05 days (35.90–42.20) vs. 31.56 days (27.1-36.11), P = 0.012; log rank].

## Discussion

The present study showed that the risk of 60-day mortality was 3 times more in malnourished patients based on the Z-score of BMI for age, 2.6 times more based on the Z-score of weight for length and 2.3 times more based on the Z-score of weight for age. The results showed no association between Z-score of height and length for age, the time of initiation of nutrition and calories and protein intake during PICU stay and the rate of mortality.

The prevalence of undernutrition based on underweighting was 41.2% in our study. In general, the prevalence of malnutrition in children admitted to PICUs has been reported as 15–57% in previous studies. It should be noted, however, that in many of these previous studies, the children had been divided into three groups, including normal nutrition, malnourished and obese. Meanwhile, obese children were excluded from the present study, which partially explains the higher percentage of malnourished children compared to previous studies.

Observational studies have shown that undernutrition is linked to mortality incidence. They have focused on all the children admitted to the PICU, or a specific group of children [2, 5, 12–20]. The results of a recent meta-analysis on 17 observational studies in the PICU showed that undernourished patients had a slightly higher risk of mortality (RD = 0.02, *P* = 0.05) [23]. In our study, this risk was fairly high, which could be due to the higher PIM2 score of the patients at the time of admission to the PICU. In addition, the incidence of undernutrition at the time of admission was relatively high in the present study, which could be because most of the children admitted to the PICU had a chronic disease that had caused undernutrition.

Although there are various methods for the anthropometric assessment of children, most studies have used the variables of height and weight, and other anthropometric indexes such as arm circumference have been less frequently used to evaluate malnutrition [1].

In the present study, the prevalence of undernutrition was 45.2% based on the Z-score of weight for age, which was similar to the prevalence of malnutrition based on the Z-score of BMI or weight/length for age. Using the weight index alone is not an accurate measure, especially in the PICU, where the possibility of edema in children is high. Nevertheless, when it is not possible to measure height or there is no data for height, the measurement of weight upon admission before the potential development of edema can be valuable.

The mortality rate was 27.8% in the present study, which is higher than the values reported by a previous study on the same subject [12, 20]. This disparity could be because the prevalence of undernutrition at admission was greater in this study than in previous studies. Furthermore, the intake of energy and protein was inadequate during hospitalization. The adequate intake of protein and energy has been shown to prevent the breakdown of respiratory and cardiac muscles in addition to skeletal muscles [24]. Furthermore, 63% of the patients started feeding after 48 h. Moreover, invasive mechanical ventilation itself may induce progressive dysfunction and atrophy of the diaphragm and respiratory muscles, which, if associated with undernutrition, increases [25]. All these factors cumulatively increased the patients’ mortality in this study. Severe undernutrition has been shown to be independently associated with higher mortality than non-severe undernutrition even with a similar PRISM score. [16].

The role of undernutrition is so important that nutritional parameters are suggested to be considered in ICU severity scoring systems [26].

Currently, it is recommended to start nutrition in the intensive care unit as early as possible. Past studies have demonstrated the positive consequences of nutritional intake in the ICU. In a study by Mehta et al. [27], the intake of at least 1.5 g of protein per kilogram of body weight was required for positive protein balance. During hospitalization in the PICU, the severity of muscle breakdown is increased by the inadequate intake of calories and protein besides the presence of a chronic disease [27]. Meanwhile, no correlation was observed between energy/protein intake and mortality in the present study. This issue can be explained by noting that only 23% of the children (n = 30) examined in this study received more than 80% of their energy/protein needs, and a larger number of cases were probably needed to find a significant relationship. Nonetheless, in a similar study conducted by Ventura et al. [10], nutritional intake was not related to mortality rate.

Of course, the importance of nutritional support during the treatment of critically ill children has been emphasized [28], especially since nutritional status often worsens during the pediatric ICU stay [29, 30].

To the best of our knowledge, this is the first cohort study in our country to specifically address the association between undernutrition and mortality in children admitted to the PICU for respiratory failure. This study had several limitations. First, it was conducted in one center and the results cannot be generalized to other centers. Second, the sample size were calculated according to nutritional status based on anthropometric indices and a larger sample size may be needed to determine the relationship between nutritional intake and the clinical outcomes. In addition, due to the restrictions imposed in the PICU, other anthropometric measurements, such as arm circumference, were not taken into consideration.

## Conclusion

Undernutrition is associated with a high mortality rate in patients with acute respiratory failure who are dependent on invasive mechanical ventilation. Weakness and atrophy of the muscles involved in respiration, both due to the presence of malnourishment and due to the damage caused by the ventilator, indicate the need of these patients for careful nutritional investigations and interventions.

## Data Availability

Data will not be made available in a public repository as we have not obtained ethical clearance to share data publicly. However, on request from corresponding author data could be provided while maintaining anonymity.
